# Psychosocial factors during the first year after a coronary heart disease event in cases and referents. Secondary Prevention in Uppsala Primary Health Care Project (SUPRIM)

**DOI:** 10.1186/1471-2261-7-36

**Published:** 2007-11-21

**Authors:** Mats Gulliksson, Gunilla Burell, Lennart Lundin, Henrik Toss, Kurt Svärdsudd

**Affiliations:** 1 Family Medicine and Clinical Epidemiology Section, Department of Public Health and Caring Sciences, Uppsala University, Uppsala, Sweden; 2 Internal Medicine Section, Department of Medical Sciences, Uppsala University Hospital, Uppsala, Sweden

## Abstract

**Background:**

A large number of studies have reported on the psychosocial risk factor pattern prior to coronary heart disease events, but few have investigated the situation during the first year after an event, and none has been controlled. We therefore performed a case-referent study in which the prevalence of a number of psychosocial factors was evaluated.

**Methods:**

Three hundred and forty-six coronary heart disease male and female cases no more than 75 years of age, discharged from hospital within the past 12 months, and 1038 referents from the general population, matched to the cases by age, sex and place of living, received a postal questionnaire in which information on lifestyle, psychosocial and quality of life measures were sought.

**Results:**

The cases were, as expected, on sick leave to a larger extent than the referents, reported poorer fitness, poorer perceived health, fewer leisure time activities, but unexpectedly reported better social support, and more optimistic views of the future than the referents. There were no significant case-referent differences in everyday life stress, stressful life events, vital exhaustion, depressive mood, coping or life orientation test. However, women reported less favourable situations than men regarding stressful life events affecting others, vital exhaustion, depressive mood, coping, self-esteem, sleep, and symptom reporting, and female cases reported the most unfavourable situation of all groups.

**Conclusion:**

In this first controlled study of the situation during the first year after a CHD event disease and gender status both appeared to be determinants of psychological well-being, with gender status apparently the strongest. This may have implications for cardiac rehabilitation programmes.

## Background

Cardiovascular disease (CVD) is the leading cause of death for men and women in the industrialized world, despite a decline in incidence [1] and mortality [2] in recent decades. CVD, and coronary heart disease (CHD) in particular, is influenced, positively or negatively, to a substantial degree, by lifestyle, and emotional and behavioural factors, in both first time event and recurrent events [3]. In recent decades psychosocial, emotional, and personality factors have come into focus [4-8].

In a number of studies psychosocial risk factors have been shown causally related to coronary heart disease incidents among men as well as among women, as summarized by Rosengren et al. [9] In these studies the psychosocial factors were measured prospectively before the incident. From a secondary prevention perspective it would be interesting to know what happens with these risk factor levels after a first event. A number of studies have addressed this question among men and women [10-16]. The findings from these studies generally show a tendency to improvement of the psychosocial situation during the first year after the event, even though the results are at variance, and most of the studies were small and uncontrolled.

The Secondary Prevention in Uppsala Primary Health Care (SUPRIM) project is an ongoing randomized controlled clinical trial in which two secondary cardiovascular prevention programmes are being evaluated in coronary artery disease patients. In this report we used baseline data from these patients within one year after the event and the corresponding data from matched referents from the general population, focussing on the psychosocial situation.

## Methods

### Study population

Patient inclusion criteria were age at most 75 years, discharged from Uppsala University Hospital after a myocardial infarction (MI) or percutaneous coronary intervention (PCI) or coronary artery bypass grafting (CABG), living in the hospital primary catchment area, referred back to the general practitioner (GP) within one year after the hospital admission, not having previously participated in similar programmes, being Swedish speaking, and being willing to participate in the study. All patients fulfilling the inclusion criteria were informed about the study during the first outpatient visit to the hospital two weeks after discharge. During a visit to the cardiology outpatient department three months after discharge the informed patients were formally invited to participate and verbal informed consent was obtained, standard requirement at the time. A written invitation letter to a baseline examination in the study was then mailed to the patients and those who accepted the invitation were eventually included. The recruitment period lasted from 1997 to 2002 and the follow-up data collection was completed in early 2007.

At the time of this report 778 consecutive patients had been considered for inclusion, of whom 287 did not fulfil the inclusion criteria, and 145 patients declined to participate, in most cases because of long distance from home to the hospital or lack of time. The remaining 346 patients (70.5% of eligible cases) agreed to participate, of whom 176 (50.9%) had been admitted for an MI, 119 (34.4%) for a CABG, and 51 (14.7%) for a PCI. Sixty-three MI patients had a PCI performed during the course of events and two had a CABG. There were no significant age, gender or diagnosis differences between participants and non-participants and no diagnosis differences between men and women. All included patients ("cases") answered a questionnaire at the baseline examination before randomisation and intervention.

All Swedish residents have a unique personal identification number, including information on date of birth and sex, stored in a population register that must be kept up-to-date by law. For each case three referents, matched by age, sex and place of living, were sampled from the register in 2002. The 1038 referents received a postal questionnaire with relevant questions from the case questionnaire. Among the cases all 346 (100%) responded and among the referents 610 (59%), altogether 956 persons. For 92% of the cases at least one of the matched referents responded, yielding 318 case-referent constellations with one case and at least one referent.

### Social and demographic data

At the time of the study more than 95% of the Swedish population in these age groups were Caucasians and about 90% were native Swedes. Information on social background and lifestyle factors was obtained from the questionnaire. For this study marital status was classified as single (including divorced or widowed) or married/cohabiting, educational level as university education or less, and smoking habits as currently smoker or not smoker. Snuff taking was classified accordingly. The participants were asked to indicate whether they had a job, had retired at normal old age retirement age (at the time of the data collection 65 years of age), or had received a disability pension. Non-retired subjects were asked whether they had been on sick leave during the past six months, and if so, for how many weeks.

### Psychosocial data

The Everyday Life Stress Scale instrument [17] was used to assess the level of self-rated stress behaviour. It consists of two major themes, time urgency/impatience and easily aroused irritation/hostility. Responses to the 20 statements were given on four-point scales (0–3), higher scores indicating more stressful reactions. Internal consistency between the 20 items is high (Cronbach's alpha = 0.90). A 5-point difference is of major clinical significance [18].

The Stressful Life Events instrument is derived from a more extensive life event scale [19]. The present version, whish comprises ten items, was previously used and validated in a Swedish study of women with cardiac disease [20]. Subjects are asked to indicate whether the stressful events asked about occurred during the past year (2), before the past year (1), or never (0). For this report the events were subdivided into those affecting the respondent and others, high scores indicating more stressful life events.

Vital exhaustion was measured with the Maastricht Questionnaire [21], also used previously in a Swedish study of women with cardiac disease [20]. Responses to the 19 items were given on three-point scales (0–2), high scores indicating a high degree of vital exhaustion. In a validation of the instrument a difference of 5 points is considered to be of major inter- or intra-individual significance [21,22].

The Depressive Mood Scale contains 20 items, selected from the Hamilton Depression Scale [23] and the Beck Depression Inventory (BDI) [24]. Both instruments are widely used, validated measures of depression severity during the past week [23,24]. The items were partially modified to achieve a standardised unidirectional response format. Possible responses were "not at all" (0), "not quite" (1), "quite well" (2), or "fully" (3), high scores indicating more depressive mood.

The Social Support Scale was originally developed by Henderson et al. [25], and later compiled into a short 30 item version by Undén and Orth-Gomer who also validated the short version [26]. In the present study, two of the four subscales were used, "availability of attachment" (AVAT), focusing on affectionately close relationships (social network quality), and "availability of social integration" (AVSI), estimating the size of the social network. AVAT has seven items, with response alternatives "yes" (1) or "no" (0), or "yes" (1), "not sufficient" (0) or "not at all" (0), yielding total score ranges of 0–7 points, high scores indicating more social integration and support. AVSI has six items, response alternatives ranging from "no one" to "more than 15 people" with a total score range of 0–30 points, high scores indicating a large social network.

The original Interpersonal Support Evaluation List (ISEL) was developed and validated by Cohen et al. [27]. It has four subscales with a total of 40 statements about the perceived availability of potential social resources. In this study a condensed 13-item version was used, with three subscales. "Appraisal" includes items on perceived availability of someone to talk with about problems. "Belonging" focuses on the availability of people to share activities with, and "Tangible" covers availability of material aid. Total score range (number of responses indicating support) was 0–13 points, high scores indicating more interpersonal support.

The Mastery Subscale of Factor Items Measuring Coping Resources [28] was used to measure coping. Subjects are asked to indicate their agreement on a four-point scale ranging from "not at all" (0) to "completely" (3) for each of the seven items, high scores indicating more coping resources.

The Life Orientation Test (LOT) was initially developed and validated by Pearlin and Schooler to assess individual differences in generalized optimism versus pessimism [29]. It contains ten items with response alternatives ranging from "I disagree strongly" (0) to "I agree strongly" (3), high scores indicating optimism.

### Quality of life

The Gothenburg Quality of Life Instrument, previously validated [30] and used in many studies, was used to measure quality-of-life aspects. For this report, the Complaint Score, the Perceived Health and the Activity Score subscales were used. The subscale Complaint Score is a list of 30 general symptoms, not intended to measure specific symptoms but rather the tendency to report symptoms. The subjects are asked to indicate what symptoms they had experienced during the past three months. In the Perceived Health subscale the subjects are asked to rate their work situation, home and family situation, fitness, mood, energy, patience, self-esteem, sleep, and well-being on seven-point interval scales ranging from "poor" (=1) to "excellent, could not be better" (=7). The Activity Score subscale contains questions on 32 specified leisure time activities and two open alternatives covering six areas. The subjects are asked to indicate which of these activities they had performed during the last two months with response alternatives "never" (0), "occasionally" (1) or "often or regularly" (2). The scores were summed across the area and to an overall activity score, high scores indicating active lifestyle.

The Ladder of Life, developed by Cantril [31] and Andrews et al. [32], is often used as an indicator of well-being. The subjects are asked to rank their perceived present well-being, what it was one year ago, and what they think it will be one year from now, on ladder-like scales ranging from the worst (=0) to the best possible situation (=9), high scores indicating a better perceived or expected well-being. The instrument was used in the NIH Post-CABG Study [33,34] and used in previous Swedish studies of subjects with CVD [35]. An validation shows that a 1-point difference is regarded as significant on the individual level [33-35]. The Research Ethics Committee at Uppsala University approved the study.

### Statistical analysis

Data were analyzed using the SAS statistical programme package [36]. The frequency of missing data in returned questionnaires was less than 2%. A power calculation was performed based on data on vital exhaustion from a controlled trial in female CHD patients and referents using the same instrument as we did [17]. Given a modest mean difference between cases and referents of 3.3 points with standard deviation 10.0, an 80% power would be obtained with a study population of 300 persons. The actual study population of 956 persons gave more than 95% power. Similar statistical power was obtained using the actual differences in activity score found in this study.

Crude differences in characteristics between the groups were tested with Student's t-test or analysis of variance for continuous data and the chi-square test for nominal or ordinal data. Differences in psychosocial and quality of life data between the groups were tested in conditional multiple linear regression or conditional ordinal logistic regression analyses adjusted for the influence of age, education, marital status and smoking habits, and adjusted means were generated using these procedures. All tests were two-tailed. To account for the many tests performed, p-values <0.005 were considered to indicate statistical significance.

## Results

### Characteristics of the study population

Seventy-six per cent of the subjects were men and mean age was 66 years, Table 1. More than half were residents of urban areas, and one third were single. On average 23% had a university education, 15% were daily smokers. Only men were snuff takers. On average 55% were old age pensioners, and 12% had disability retirement benefits. Based on the 312 subjects who could be on sick leave 26% of the referents were on sick leave versus 68.8% of the female cases and 61.3% of the male cases.

**Table 1:** Characteristics of the study population.

	Women				Men					
				
	Cases		Referents		Cases		Referents		Sex differences	Case-reference differences
				
	n	mean or %	n	mean or %	n	mean or %	n	mean or %	p	p
Age, years	82	67.5	149	66.9	264	64.9	461	65.1	0.0003	0.97
Urban dwellers, %	56	68.3	106	71.1	155	58.7	272	59.0	0.0022	0.77
Single, %	29	35.4	64	43.2	33	12.6	94	20.7	<0.0001	0.0041
University education, %	17	20.7	41	27.7	53	20.3	115	25.1	0.56	0.0634
Daily smokers, %	12	14.8	18	12.2	34	13.0	74	16.3	<0.0001	<0.0001
Snuff takers, %	0	0	1	0.7	26	10.0	57	12.5	<0.0001	0.65
Old age pension, %	48	58.5	99	66.9	120	45.5	255	55.7	0.0016	0.0034
Disability pension, %	16	19.5	15	10.1	31	11.7	51	11.1	0.38	0.21
Sick-listed										
Among all, %	15	19.2	15	10.1	80	31.5	47	10.3	0.12	<0.0001
Among eligible, %	11	68.8	9	26.5	68	61.3	39	25.8	0.68	<0.0001

Characteristics of the study population. P-values refer to differences between men and women and between cases and referents in conditional analyses and p < 0.005 were regarded as statistically significant.

### Psychosocial measures

After adjustment for the influences of age, education, marital status and smoking habits, there were no differences between the groups regarding everyday life stress and stressful life events, Table 2. Events affecting others were more often reported by women than men, as were vital exhaustion (p < 0.0001) and depressive mood scores (p < 0.001), while there were no significant differences between cases and referents for these variables. Cases reported higher social support scores than their referents. Men reported higher coping scores than women but with no significant differences between cases and referents. Women reported less optimism then men, and also the lowest optimism scores of all groups.

**Table 2:** Psychosocial measures.

		Women		Men		Sex differences	Case-referent differences
					
	Score range	Cases	Referents	Cases	Referents	p	p
N		82	149	264	461		
Everyday Life Stress	0–60	17.5	18.3	18.9	18.6	0.75	0.92
Stressful Life Events	0–20	4.0	3.8	3.7	3.7	0.06	0.92
Affecting own person	0–6	1.0	0.8	1.4	1.1	0.15	0.057
Affecting others	0–14	3.0	3.0	2.3	2.6	<0.0001	0.055
Vital exhaustion	0–38	16.8	15.6	12.2	12.7	<0.0001	0.48
Depressive mood	0–60	21.4	20.0	16.8	17.9	<0.001	0.31
Social support Scale	0–39	23.0	21.3	23.4	22.0	0.32	0.0001
Availability of attachment (AVAT)	0–9	8.3	7.8	7.9	7.6	<0.05	0.0008
Availability of social integration (AVSI)	0–30	14.6	13.5	15.5	14.3	0.07	0.0007
Interpersonal support (ISEL)	0–39	29.1	27.7	28.7	28.0	0.81	0.0085
Appraisal	0–15	10.5	10.2	10.4	10.4	0.74	0.28
Belonging	0–15	11.6	10.9	11.3	10.8	0.45	0.0071
Tangible	0–9	7.0	6.7	7.0	6.8	0.92	0.09
Coping	0–21	14.5	14.2	15.7	15.3	<0.0001	0.054
Optimism (LOT)	0–30	18.8	19.1	20.0	19.8	0.0046	0.44

Psychosocial scale mean scores among female and male cases and referents, adjusted for the influence of age, education, marital status and smoking habits in conditional analyses. p < 0.005 were regarded as statistically significant.

### Quality of life measures

Quality of life data measured as well-being and activity score adjusted for the influence of age, education, marital status and smoking habits are shown in Table 3. Women reported a higher total well-being score then men. However, there were no significant differences between cases and referents or between men and women regarding work situation, home and family situation, mood, energy and patience. Women reported significantly lower self-esteem, sleep, fitness and perceived health than men. Cases reported lower scores for fitness and perceived health than referents.

**Table 3:** Well-being and leisure time activity.

		Women		Men		Sex differences	Case-referent differences
					
	Score range	Cases	Referents	Cases	Referents	p	p
N		82	149	264	461		
Well-being score	9–63	46.3	49.9	46.1	48.9	0.0001	0.11
Work situation	1–7	4.7	4.8	4.7	5.0	0.28	0.0162
Home and family situation	1–7	5.6	5.4	5.7	5.8	0.21	0.0091
Mood	1–7	5.5	5.8	5.8	5.8	0.18	0.10
Energy	1–7	5.0	5.4	5.5	5.6	0.0264	0.09
Patience	1–7	5.6	5.8	5.6	5.8	0.41	0.0184
Self-esteem	1–7	4.7	5.0	5.6	5.5	<0.0001	0.70
Sleep	1–7	4.5	5.1	5.6	5.6	<0.0001	0.19
Fitness	1–7	4.0	4.5	4.5	4.8	0.0049	<0.0008
Perceived health	1–7	4.5	5.1	5.0	5.3	0.0021	0.0001
Activity score	0–64	21.8	24.4	26.1	29.4	<0.0001	<0.0001
Home indoor activities	0–10	4.6	5.1	4.8	5.5	0.19	0.0002
Home outdoor activities	0–8	1.9	2.3	4.5	4.8	<0.0001	0.0423
Physical activities	0–14	4.0	4.4	5.0	5.7	<0.0001	0.0061
Pleasure	0–16	4.1	4.7	4.5	5.3	0.0106	<0.0001
Social activities	0–8	4.7	5.2	4.8	5.3	0.82	0.0003
Clubs and associations	0–8	2.0	2.4	2.0	2.4	0.48	0.0125

Well-being and Activity score among female and male cases and referents after adjustment for the influence of age, education, marital status and smoking habits in conditional analyses. p < 0.005 were regarded as statistically significant.

Women reported lower total activity, home outdoor activity, and physical activity scores than men. Cases reported lower total activity, home indoor activity, pleasure activity, and social activity scores than referents.

Women reported more complaint score symptoms (10.2 95%CI 9.2–11.2 for female referents and 10.4, 95%CI 9.3–11.4 for female cases) than men (8.2, 95%CI 7.7–8.8 for male referents and 8.1, 95%CI 7.4–8.8 for male cases). The difference between men and women was significant (p < 0.0001) but not between cases and referents.

**Figure 1:**
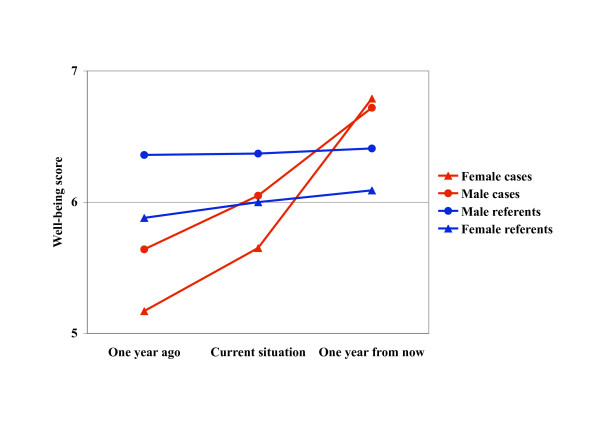
**Past, present time and future expectations**. The Ladder of Life, expressing self-rated general life situation a year ago, presently and a year from now. The symbols refer to the time point given along the horizontal axis.

The rating of the general life situation a year ago, today, and what it is expected to be a year from now, is shown in Figure 1. Referents reported a small but non-significant change over the two years, whereas cases started on a low level and had a substantial increase of their life situation scores ending up with a higher score than referents. Especially female cases had a remarkable increase. The difference in slope between cases and referents was significant (p < 0.005), but not the difference between women and men.

All results regarding psychosocial and quality of life measures were independent of diagnostic group and time from discharge from hospital to questionnaire response.

## Discussion

Cases were old age pensioners or on sick leave to a larger extent than referents, had poorer fitness, poorer perceived health and lower activity scores, but better social support, and more optimistic views of the future. There were no significant case-referent differences in everyday life stress, stressful life events, vital exhaustion, depressive mood, coping or life orientation test. There were some interesting sex differences, such as stressful life events affecting others, vital exhaustion, depressive mood, coping, optimism (LOT), self-esteem, sleep, perceived health, total activity, home outdoor activity, physical activity, and symptom reporting where women generally had worse situations than men and female cases had the worst situation of all.

The response rate was moderate among the referents, approximately 60%. However, within the matched quadruples more than 90% had at least one referent responder, and the result of the matching procedure was satisfactory. The potential bias caused by a differential number of responding referents in the matched quadruples was handled by conditional analyses, in which cases were compared only with their own referents. The number of referents per case is in this type of analysis of minor importance as long as there is at least one, since randomly sampled referents per case by definition are interchangeable. The frequency of missing data in returned questionnaires was low. Some of the questionnaires have been validated in their English version and some in their Swedish version. However, all questionnaires have been used extensively in previous studies. Moreover, in our study measurements among the cases were compared with those of matched referents. We have therefore no reason to believe that the data are affected by selection or other bias to such an extent that the conclusions have been affected. The limitations of the study include its cross-sectional nature, which limits the interpretations of cause and effect relationships.

The results regarding sick leave, perceived health, fitness, activity, and complaint score were all in accordance with expectations based on interpretations of findings in previous studies [10,12,14], even though these studies were uncontrolled. The cases generally had less favourable situations than the referents with more objective and subjective illness, and more symptom reporting.

Unexpectedly, no significant differences between cases and referents were found with respect to everyday life stress (a measure of self-rated stress behaviour), stressful life events, vital exhaustion or depressive mood. The proportion of individuals with a clinically recognisable depression is somewhat difficult to assess. According to Beck's and Hamilton's criteria a score of 20 or more indicates depression. In this case that would mean half of the study population, cases as well as referents are depressed. This is unlikely and the cut-off level might be affected by cultural factors. Estimated from one of our databases about 2% of the Swedish population have a clinical depression, which in this case would mean that also cases are depressed in approximately 2%. However, the proportion with a sub-clinical depression may by larger or much larger.

Previous research indicates that severe life events may be more common in CHD cases [6,9], although stressful life events as a CHD risk factor is controversial [8]. Even though the effects of stressful life events are supposed to be long-term, we found no significant differences between cases and referents the way we measured it within the first year after the incident. A possible explanation might be that the referent group included persons with a past history of CHD or other illnesses and therefore were more similar to the cases than a "healthy" group would have been. We have no data on the referents' medical history. However, the referents were sampled from the general population and the proportion of CHD cases in the group might be expected to be 5% or less. The probability that this proportion of CHD cases caused the non-significant case-referent difference in our study is small.

Another possibility might be that differences in marital status between men and women and cases and referents have affected the result. However, such differences were accounted for in the analyses. To our knowledge no other studies have addressed these issues. The large study population and the high statistical power of the present study indicate that it is unlikely that the differences were small due to chance. It is much more likely that they mirror a reality.

The evidence for stress as a CHD risk factor is more consistent [8,9]. The questionnaire on everyday life stress in our study had no specified time limit, but the wording is such that it can be interpreted as dealing with the present situation. The vital exhaustion questionnaire in this study covers the past three months and the depression questionnaire the past week. The cases may have reduced their stress related behaviour after the event, perhaps because they feel the need to be more relaxed, or they may withdraw from stress-provoking situations. It may be that the situation regarding stress, depression or vital exhaustion was quite different before the event, while during the first year after the event the cases' situation was more similar to the referents. This issue has not been addressed specifically in previous studies, but some support for an improvement of the physical and social function and mental health (depression) over time was reported by Kristofferzon et al. [14]. Again, the sample size and statistical power favour that our finding mirror a reality.

The cases reported a better social support situation than referents, significant for the Social Support Scale and the two subscales. This was un unexpected finding but may have the same explanation as stress related behaviour above, i.e., things may have calmed down during the first year after the event, the family and other social support providers may have increased their support as a result of the event. It may also reflect the short hospital based rehabilitation programme preceding the present programme and offered to the cases in the sample, in which family members were actively involved. This may to some extent have improved their psychological well-being, as well as involved the families in the rehabilitation. The possible change of social support over time after a CHD event has not been addressed explicitly previously, even though some evidence of perceived support have been presented [37]. We have no reason to believe that our findings are caused by chance.

The Ladder of Life instrument offers strong evidence that the cases' life situation changed dramatically from pre-event to post-event. This change lends support to our speculation that several of the psychosocial measures we used may have improved considerably from before to after the event and reached or even surpassed the referent level.

There were major sex differences in the responses. Female referents and female cases scored significantly worse on complaints (physical and psychological symptoms), depressive mood, coping, stressful life events affecting others, optimism (LOT) and vital exhaustion than all men. This is in accordance with other studies showing that women tend to report (significantly) more health problems than do men after a CHD event [10-16]. Quality of life, assessed by the Ladder of Life, was significantly lower in both female cases and female referents as compared to men. Male referents rated the best quality of life, and female cases the worst. These results are supported by Agewall et al. who found that self-assessed quality of life after an MI was significantly lower in women than among men despite similar age, treatment and haemodynamic and laboratory data [16].

## Conclusion

In conclusion, the results indicate that both sex and disease status are determinants of psychological well-being after a CHD event. However, sex seems to be the stronger determinant, since women generally do worse than men, and the high risk person in terms of low psychological well-being is the female cardiac case. Readily measured demographic and psychosocial risk factor data may assist in the identification of post-coronary event patients who are at increased risk of poor clinical outcomes. Prevention efforts and cardiac rehabilitation should be targeted to those with increased psychosocial burden, specifically women who experience greater disparities in recovery from cardiac events.

## Competing interests

The author(s) declare that they have no competing interests.

## Authors' contributions

All authors participated in designing the study. MG and KS obtained and edited additional register data. MG, GB and KS performed the analysis and drafted the manuscript. All authors participated in the discussions of the draft outline and contributed later with text revisions, table revisions and figure revisions. All authors have seen and approved the final version of the manuscript.

## Pre-publication history

The pre-publication history for this paper can be accessed here:


